# Association between Long COVID and neuropsychological outcomes in a Brazilian Cohort: preliminary results

**DOI:** 10.1002/alz.093084

**Published:** 2025-01-03

**Authors:** Joana Emilia Senger, Luiza Santos Machado, Maiele Dornelles Silveira, Ana Paula Bornes da Silva, João Pedro Ferrari‐Souza, Marco Antônio de Bastiani, Guilherme Povala, Wyllians Vendramini Borelli, Guilherme Bastos de Mello, João Pedro Uglione da Ros, Arthur Viana Jotz, Matheus Fakhri Kadan, Graciane Radaelli, Tharick A. Pascoal, Cristina Sebastião Matushita, Ricardo Benardi Soder, Artur Francisco Schumacher‐Schuh, Diogo O. Souza, Mychael V. Lourenco, Daniele de Paula de Paula Faria, Artur Martins Coutinho, Jaderson Costa da Costa, Débora Guerini de Souza, Eduardo R. Zimmer

**Affiliations:** ^1^ Federal University of Rio Grande do Sul, Porto Alegre, Rio Grande do Sul Brazil; ^2^ Universidade Federal do Rio Grande do Sul, Porto Alegre, Rio Grande do Sul Brazil; ^3^ Federal University of Rio Grande do Sul, Brazil, Porto Alegre, RS Brazil; ^4^ University of Pittsburgh, Pittsburgh, PA USA; ^5^ Memory Center, Hospital Moinhos de Vento, Porto Alegre, RS Brazil; ^6^ Pontifícia Universidade Católica do Rio Grande do Sul, Porto Alegre, Rio Grande do Sul Brazil; ^7^ Lutheran University of Brazil, Canoas, Rio Grande do Sul Brazil; ^8^ Federal University of Rio Grande do Sul, Porto Alegre, RS Brazil; ^9^ Brain Institute, RS, Porto Alegre, Rio Grande do Sul Brazil; ^10^ Brain Institute of Rio Grande do Sul (BraIns), PUCRS, Porto Alegre, RS Brazil; ^11^ Pontifical Catholic University of Rio Grande do Sul, PORTO ALEGRE, RIO GRANDE DO SUL Brazil; ^12^ HCPA, Porto Alegre, Rio Grande do Sul Brazil; ^13^ Universidade Federal do Rio Grande do Sul, Porto Alegre Brazil; ^14^ Federal University of Rio de Janeiro, Rio de Janeiro, Rio de Janeiro Brazil; ^15^ USP, São Paulo Brazil; ^16^ University of São Paulo Medical School, São Paulo, São Paulo Brazil; ^17^ Brain Institute of Rio Grande do Sul ‐ Pontifícia Universidade Católica do Rio Grande do Sul, Porto Alegre, Rio Grande do Sul Brazil

## Abstract

**Background:**

COVID‐19 pandemic has brought long‐lasting social, emotional, and cognitive consequences. Long COVID is characterized by a myriad of symptoms and complications that persist long after the infection, including cognitive decline and mental health impairment. This study aims to investigate depressive symptoms and cognitive performance stratified by sex and group in adults with and without long COVID.

**Method:**

Community‐dwelling individuals from Porto Alegre, Brazil, were divided into control and long COVID groups. We recruited 49 individuals, aged >50 years. Individuals were evaluated with the mini mental state examination (MMSE), Clock Drawing Test, Trail Making Test (TMT‐B), Wechsler Memory Scale (WMS‐R) and PHQ‐9 scale of depressive symptoms. A regression model was conducted stratified by sex, age and group. PHQ‐9 was used as an interaction of the same variables. Data were analyzed using *R*, considering p‐value < 0.05.

**Result:**

A total of 49 individuals (59.8 ± 8.06 mean years of age, 63.3% females), having high education (>11 years of study) were included. The control group was comprised of 18 individuals (36.73%) and the long COVID group had 31 individuals (63.26%). The Long COVID group had a worse performance in WMS‐A (p <0.011) and WMS‐B (p <0.03) in the immediate test and WSM‐B recall (p <0.02). The PHQ‐9 test differed between groups, with long COVID individuals presenting depressive symptoms (p <0.003). We also found an interaction of long COVID and PHQ‐9 impacting on the TMT‐ B test (p <0.03).

**Conclusion:**

Preliminary results showed that long COVID induced impairment in executive functions, specifically in working memory and episodic memory scores. Additionally, there was an impact of depressive symptoms in the relationship between group and TMT‐B scores. Participants with depressive symptoms had worsened cognitive performance on the regression analysis. A larger sample should corroborate these findings and provide further insights into the long COVID cognitive impact.

